# The Ratio of Red Blood Cell Distribution Width to Albumin Is Correlated With All-Cause Mortality of Patients After Percutaneous Coronary Intervention – A Retrospective Cohort Study

**DOI:** 10.3389/fcvm.2022.869816

**Published:** 2022-05-24

**Authors:** Yingbei Weng, Yangpei Peng, Yuxuan Xu, Lei Wang, Bosen Wu, Huaqiang Xiang, Kangting Ji, Xueqiang Guan

**Affiliations:** Department of Cardiology, The Second Affiliated Hospital and Yuying Children’s Hospital of Wenzhou Medical University, Wenzhou, China

**Keywords:** all-cause mortality, coronary artery disease, percutaneous coronary intervention, the ratio of red blood cell volume distribution width, albumin

## Abstract

**Objectives:**

The purpose of this study was to investigate the independent effect of the ratio of red blood cell distribution width (RDW) to albumin (RA) on all-cause mortality in patients after percutaneous coronary intervention (PCI).

**Methods:**

Clinical data were obtained from the Multiparameter Intelligent Monitoring in Intensive Care-III (MIMIC-III) database version 1.4 and the database of Second Affiliated Hospital and Yuying Children’s Hospital of Wenzhou Medical University. We used the MIMIC-III database for model training, and data collected from the Second Affiliated Hospital of Wenzhou Medical University for validation. The primary outcome of our study was 90-day mortality. Cox proportional hazards regression model was used to estimate hazard ratio (HR) for the association between RA and all-cause mortality in patients after PCI. Pearson correlation analysis was conducted to assess the relationship between RA and Gensini score or cardiac troponin I (cTnI).

**Results:**

A total of 707 patients were eligible in MIMIC-III database, including 432 males, with a mean age of 70.29 years. For 90-day all-cause mortality, in the adjusted multivariable model, the adjusted HRs [95% confidence intervals (CIs)] for the second (RA: 3.7–4.5 ml/g) and third (RA >4.5 ml/g) tertiles were 2.27 (1.11, 4.64) and 3.67 (1.82, 7.40), respectively, compared to the reference group (RA <3.7 ml/g) (*p* < 0.05). A similar relationship was also observed for 30-day all-cause mortality and 1-year all-cause mortality. No significant interaction was observed in subgroup analysis. Receiver operating characteristic (ROC) curve analysis proved that the ability of RA to predict the 90-day mortality was better than that of RDW or albumin alone. The correlation coefficient between Gensini score and RA was 0.254, and that between cTnI and RA was 0.323.

**Conclusion:**

RA is an independent risk factor for all-cause mortality in patients after PCI. The higher the RA, the higher the mortality. RA has a good predictive ability for all-cause mortality in patients after PCI, which is better than RDW or albumin alone. RA may be positively correlated with the severity of coronary artery disease (CAD) in patients with CAD.

## Introduction

Coronary artery disease (CAD) is an atherosclerotic disease dominated by inflammation (1, 2). CAD was reported to be one of the most common causes of death in developed and developing countries (3, 4). It is estimated that 18.2 million Americans aged 20 years or older suffered from CAD between 2013 and 2016, and the total CAD prevalence was about 6.7%, representing a substantial increase compared to previous years. According to 2017 United States mortality data, 365,914 Americans succumbed to CAD (5). Percutaneous coronary intervention (PCI) has already been one of the most commonly used interventions for CAD (6). Although reasonable and timely PCI can improve the prognosis of CAD patients, CAD still has a high rate of disability and mortality. Therefore, this calls for effective and convenient prognostic biomarkers that can help doctors to make medical decisions and identify high-risk patients.

Atherosclerotic lesions can be described as an inflammatory disease characterized by a series of highly specific cellular and molecular responses (1, 7–10). Red blood cell distribution width (RDW) is one of the parameters measured in blood routine testing. As an easily accessible marker of systemic inflammation, RDW has been identified as a new prognostic factor for many diseases, including cardio-cerebrovascular diseases and various infectious diseases (11). Moreover, a low serum albumin level is strongly negatively correlated with the risk of death from cardiovascular diseases (12). Recent studies have shown that the ratio of RDW to albumin (RA) is associated with the prognosis of a variety of diseases, including aortic aneurysm, diabetic retinopathy, and shock (13–15). However, it is unknown whether combining RDW with albumin can more effectively predict the prognosis of patients after PCI.

In this study, real world data were used to assess the association between RA and outcome of patients after PCI.

## Materials and Methods

### Study Population

All data were obtained from the databases of Multiparameter Intelligent Monitoring in Intensive Care (MIMIC-III) and our hospital (the Second Affiliated Hospital of Wenzhou Medical University).

MIMIC-III database version 1.4 is a large single-centered database containing the data of more than 50,000 adult patients admitted to the intensive care unit (ICU) in different hospitals. MIMIC-III database was approved by the Institutional Review Boards of the Beth Israel Deaconess Medical Center and the Massachusetts Institute of Technology.

With regard to the data obtained from our hospital, the Institute of Institutional Research and Ethics of the Second Affiliated Hospital of Wenzhou Medical University approved the experiments involving human subjects. The ethical review approval number is 2021-K-71-01. Informed consent was not required as the data were anonymous.

Subjects to be included had to meet the following criteria: (1) patients after PCI; patient records with PCI treatment were retrieved from the MIMIC-III database using Structured Query Language (SQL). In our hospital, we enroll patients by reviewing their coronary angiography records and surgical records; (2) adult patients (age ≥18 years). The exclusion criteria were as follows: (1) more than 10% of the patient’s individual data is missing; (2) when there are outliers in patient data: the value exceeds mean ± 3 standard deviation (SD); (3) RDW or albumin data are missing. The first ICU admission records were used when patients had multiple hospitalizations.

### RA Assessment

Blood samples were collected from subjects within 24 h after admission to the ICU. RDW and albumin were measured by medical instruments. RDW was expressed as percent (%) and albumin was expressed as g/dl. RA was calculated as the ratio of RDW to albumin.

### Assessment of Other Covariates

Structured query language was used to extract the data recorded on the first day of admission from the database, including demographic data, basic vital signs, comorbidities, basic laboratory parameters, and pre-treatment scoring systems. Demographic information included race, gender, and age. Basic vital signs included blood pressure, respiratory rate, heart rate, body temperature, and saturation of pulse oxygen (SpO_2_). Complications included hypertension, arrhythmia, heart valve disease, congestive heart failure, pulmonary circulation disorders, chronic pulmonary disease, peripheral vascular disease, coagulopathy, electrolyte disorder, nervous system diseases, hypothyroidism, and obesity. Laboratory parameters included RDW, albumin, hemoglobin, hematocrit, platelet, white blood cell count, creatinine, urea nitrogen, chloride, sodium, bicarbonate, and anion gap. In addition, simplified acute physiology score II (SAPSII) and acute physiology score III (APSIII) were included. During variable selection, variables with missing values ≥40% were directly excluded.

Furthermore, Gensini score and cardiac troponin I (cTnI) were additionally extracted from the database of our hospital. The Gensini score was used to assess the severity of coronary artery stenosis, as shown in Supplementary Table 1. The Gensini score was the sum of individual coronary segment scores (16), separately defined by two independent doctors.

### Outcomes

In this study, the primary outcomes were 90-day mortality, while the secondary outcomes were 30-day mortality, 1-year mortality, length of stay in ICU, and length of hospital stay. The observation time was from the patient’s first admission at the hospital until death.

### Statistical Analyses

The included patients were divided into three groups according to the RA level, and their baseline characteristics were described. Smooth curve fitting and Kaplan–Meier curves were generated. Cox proportional hazard regression was used to evaluate the association between all-cause mortality and RA, and the results were shown as a hazard ratio (HR) with a 95% confidence interval (CI) (17). Three multivariate analysis models were established for each outcome. In model 1, the covariates were not adjusted; in model 2, the adjusted covariates included race, gender, and age; and in model 3, we further adjusted for SpO_2_, respiratory rate, heart rate, diastolic blood pressure, temperature, congestive heart failure, heart valve disease, nervous system diseases, pulmonary circulation disorders, hypertension, chronic pulmonary disease, hypothyroidism, obesity, coagulopathy, white blood cell count, serum chloride, bicarbonate, platelet, hematocrit, and anion gap. The choice of covariates was based on the estimated value of impact >10% (18), and the low RA level group (RA <3.7 ml/g) was set as the reference group. Subgroup analysis was used to determine the influence of RA on 90-day all-cause mortality in different subgroups. Receiver operating characteristic (ROC) curve analysis was performed to predict the prognostic efficiency of the RA. Moreover, we compared the RA with other prognostic indicators to verify the superiority of RA in predicting the prognosis of such patients. The RA value was analyzed by Pearson correlation analysis using Gensini score and cTnI, respectively.

All analyses were conducted using R software (Version 3.6.1^1^). All *p*-values were two-sided, and *p* < 0.05 was considered statistically significant.

## Results

### Characteristics of Patients

**FIGURE 1:**
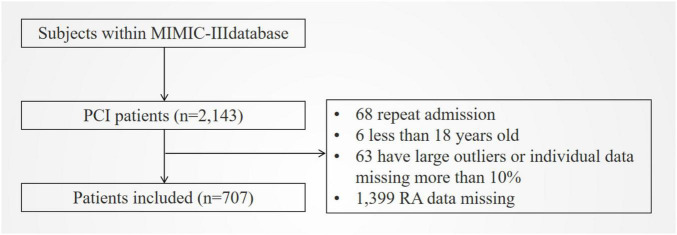
Flowchart of included patients. MIMIC III, Multiparameter Intelligent Monitoring in Intensive Care Database III; PCI, percutaneous coronary intervention; RA, the ratio of red cell volume distribution width to albumin.

In total, 707 patients were included (Figure 1). Table 1 shows the baseline characteristics of patients grouped by RA level. As seen in the table, the higher RA group was older, had lower blood pressure, faster heart rate, higher SAPSII and APSIII scores (*p* < 0.001), and a higher chance of complications, including congestive heart failure, heart valve disease, electrolyte disorder, peripheral vascular disease, coagulopathy, and chronic pulmonary disease (all *p* < 0.01), compared to the lower group. They also had higher levels of RDW, serum creatinine, serum urea nitrogen, and anion gap, as well as lower levels of albumin, platelet, hemoglobin, hematocrit, serum sodium, and bicarbonate (all *p* < 0.05). As shown in the table, the higher RA group had higher all-cause mortality, longer ICU stay, and shorter overall survival (*p* < 0.001). Moreover, the baseline characteristics of patients grouped by 90-day all-cause mortality are shown in Supplementary Table 2. In addition, Supplementary Table 3 shows the baseline characteristics of patients in the cohort of our hospital. The higher RA group was older, had a faster heart rate, and had a higher risk of congestive heart failure, arrhythmia, and cardiogenic shock. In addition, the higher the RA, the higher the Gensini score and the longer the length of hospital stay.

**TABLE 1:** Baseline characteristics of the study population.

Characteristics	RA level, ml/g			*p-*Value
	<3.7 (*n* = 233)	3.7–4.5 (*n* = 238)	>4.5 (*n* = 236)	
**Clinical parameters**				
Age, years	61.7 ± 13.3	71.2 ± 12.9	71.8 ± 12.5	<0.001
Sex, *n* (%)				<0.001
Male	176 (75.5)	136 (57.1)	120 (50.8)	
Female	57 (24.5)	102 (42.9)	116 (49.2)	
Ethnicity, *n* (%)				0.800
White	142 (60.9)	153 (64.3)	154 (65.3)	
Black	12 (5.2)	9 (3.8)	12 (5.1)	
Other	79 (33.9)	76 (31.9)	70 (29.7)	
**Vital signs**				
SBP, mmHg	115.0 ± 14.3	111.5 ± 14.8	109.8 ± 16.3	<0.001
DBP, mmHg	64.3 ± 10.1	58.6 ± 8.7	56.2 ± 9.9	<0.001
MAP, mmHg	80.7 ± 9.6	76.6 ± 9.2	74.7 ± 9.8	<0.001
Heart rate, beats/min	77.4 ± 14.8	79.7 ± 13.1	83.6 ± 16.1	<0.001
Respiratory rate, times/min	18.2 ± 3.3	18.8 ± 3.5	18.7 ± 3.9	0.189
Temperature, °C	36.8 ± 0.5	36.8 ± 0.6	36.8 ± 0.7	0.992
SpO_2_, %	97.2 ± 1.7	97.4 ± 1.6	97.1 ± 3.4	0.432
**Comorbidities**				
Congestive heart failure, *n* (%)	74 (31.8)	125 (52.5)	149 (63.1)	<0.001
Arrhythmia, *n* (%)	102 (43.8)	106 (44.5)	117 (49.6)	0.390
Heart valve disease, *n* (%)	23 (9.9)	50 (21.0)	53 (22.5)	<0.001
Peripheral vascular disease, *n* (%)	12 (5.2)	23 (9.7)	41 (17.4)	<0.001
Hypertension, *n* (%)	129 (55.4)	151 (63.4)	135 (57.2)	0.174
Pulmonary circulation disorders, *n* (%)	11 (4.7)	24 (10.1)	22 (9.3)	0.070
Chronic pulmonary disease, *n* (%)	37 (15.9)	51 (21.4)	68 (28.8)	0.003
Coagulopathy, *n* (%)	13 (5.6)	20 (8.4)	38 (16.1)	<0.001
Electrolyte disorder, *n* (%)	23 (9.9)	29 (12.2)	68 (28.8)	<0.001
Nervous system diseases, *n* (%)	14 (6.0)	13 (5.5)	10 (4.2)	0.677
Hypothyroidism, *n* (%)	14 (6.0)	22 (9.2)	19 (8.1)	0.416
Obesity, *n* (%)	10 (4.3)	10 (4.2)	10 (4.2)	0.999
**Laboratory parameters**				
RDW, %	13.2 ± 0.7	14.0 ± 1.0	15.8 ± 2.2	<0.001
Albumin, g/dl	4.0 ± 0.3	3.5 ± 0.3	2.9 ± 0.5	<0.001
White blood cell count, 10^9^/L	11.0 ± 3.6	10.9 ± 4.2	10.7 ± 5.4	0.783
Platelet, 10^9^/L	229.3 ± 61.4	205.5 ± 82.2	191.5 ± 88.3	<0.001
Hemoglobin, g/dl	12.4 ± 1.7	10.8 ± 1.7	9.5 ± 1.8	<0.001
Hematocrit, %	35.7 ± 4.7	31.4 ± 4.7	28.2 ± 5.1	<0.001
Serum creatinine, mg/dl	1.0 ± 0.5	1.2 ± 1.1	1.7 ± 1.8	<0.001
Serum urea nitrogen, mg/dl	18.0 ± 12.3	24.3 ± 14.4	31.0 ± 22.3	<0.001
Serum chloride, mg/dl	101.4 ± 4.6	102.4 ± 4.7	102.4 ± 6.2	0.070
Serum sodium, mg/dl	136.3 ± 3.7	136.2 ± 3.8	135.1 ± 4.9	0.003
Bicarbonate, mg/dl	22.7 ± 3.3	21.8 ± 4.1	19.8 ± 4.8	<0.001
Anion gap, mg/dl	13.2 ± 2.8	13.4 ± 2.7	13.9 ± 3.4	0.027
**Scoring systems**				
SAPSII	28.5 ± 12.3	35.4 ± 12.1	42.5 ± 14.3	<0.001
APSIII	35.2 ± 16.3	41.2 ± 16.7	51.8 ± 21.6	<0.001
**Mortality**				
30-day	10 (4.3)	24 (10.1)	43 (18.2)	<0.001
90-day	12 (5.2)	32 (13.4)	65 (27.5)	<0.001
1 year	19 (8.2)	50 (21.0)	97 (41.1)	<0.001
Length of stay in ICU	3.2 ± 4.2	4.5 ± 7.9	6.8 ± 8.7	<0.001
Survival time	895.9 ± 386.8	879.4 ± 636.9	713.0 ± 734.2	0.001

*RA, the ratio of red cell volume distribution width to albumin; SBP, systolic blood pressure; DBP, diastolic blood pressure; MAP, mean arterial pressure; RDW, red cell volume distribution width; SAPSII, simplified acute physiology score II; APSIII, acute physiology score III; ICU, intensive care unit.*

### Association Between RA and Outcomes

**FIGURE 2:**
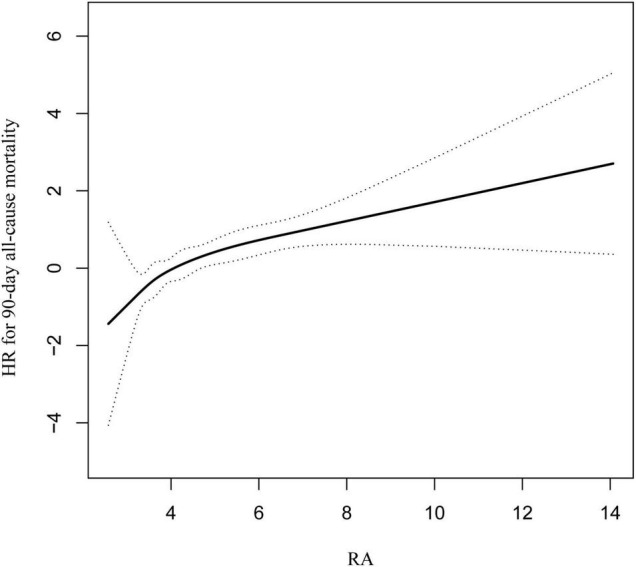
Curve fitting of RA and 90-day all-cause mortality in patients with coronary heart disease, who had undergone PCI.

To demonstrate linearity of RA and 90-day all-cause mortality of patients after PCI, smooth curve fitting was performed (Figure 2). After adjusting for possible covariates, the linear relationship was observed. In addition, smooth curve fitting was also performed for 30-day and 1-year all-cause mortality and the same relationship was observed (Supplementary Figures 1, 2).

**FIGURE 3:**
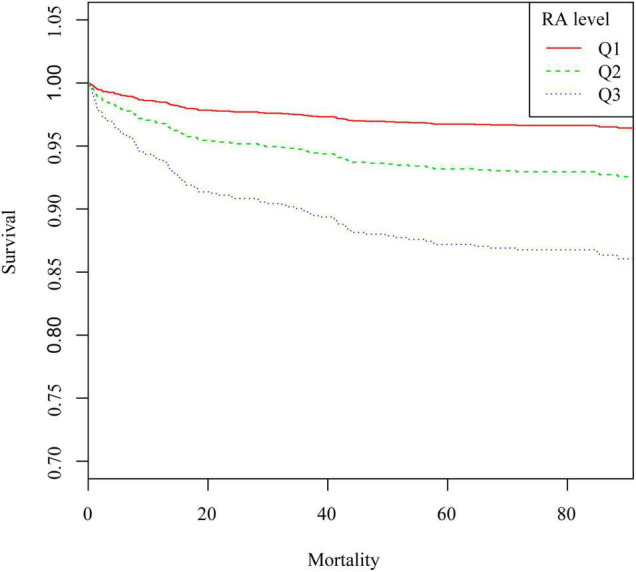
Kaplan–Meier survival curve of 90-day all-cause mortality in patients after PCI. Q1, RA < 3.7 ml/g; Q2, RA was 3.7–4.5 ml/g; Q3, RA > 4.5 ml/g.

Figure 3 shows the 90-day survival curves of patients after PCI, stratified by the tertiles of RA. The figure shows significantly lower cumulative survivals with higher RA tertiles. Similar correlations were also observed in 30-day and 1-year survival curves (Supplementary Figures 3, 4).

Table 2 shows the results of the relationship between RA and each outcome indicator. With regard to the 90-day all-cause mortality, in the unadjusted model, the HRs (95% CIs) for the second (RA: 3.7–4.5 ml/g) and third (RA >4.5 ml/g) tertiles were 2.73 (1.41, 5.30) and 6.03 (3.26, 11.17), respectively, compared to the reference group (RA <3.7 ml/g) (*p* < 0.05). This association remained statistically significant even after adjusting for race, gender, age, SpO2, respiratory rate, heart rate, diastolic blood pressure, temperature, congestive heart failure, heart valve disease, nervous system diseases, pulmonary circulation disorders, hypertension, chronic pulmonary disease, hypothyroidism, obesity, coagulopathy, white blood cell count, serum chloride, bicarbonate, platelet, hematocrit, and anion gap. The adjusted HRs (95% CIs) for the second (RA: 3.7–4.5 ml/g) and third (RA >4.5 ml/g) tertiles were 2.27 (1.11, 4.64) and 3.67 (1.82, 7.40), respectively, compared to the reference group (RA <3.7 ml/g) (*p* < 0.05). When analyzing RA as a continuous variable, the HRs (95% CIs) in the three models were 1.36 (1.24, 1.49), 1.48 (1.31, 1.67), and 1.43 (1.24, 1.65), respectively (all *p* < 0.001). A similar relationship was also observed for the outcome of 30-day or 1-year all-cause mortality. Furthermore, the linear regression results of the relationship between RA and length of hospital stay or ICU stay were also expressed. It showed that the beta values (95% CIs) for the length of ICU stay in the three models were 1.15 (0.70, 1.60), 1.17 (0.70, 1.63), and 0.89 (0.43, 1.36), respectively (all *p* < 0.001). The β values (95% CIs) for the length of hospital stay in the three models were respectively 1.80 (1.21, 2.39), 1.82 (1.21, 2.43), and 1.45 (0.84, 2.06).

**TABLE 2:** Hazard ratio (HR) [95% confidence intervals (CIs)] for all-cause mortality across groups of ratio of red blood cell distribution width (RDW) to albumin (RA) level.

RA level, ml/g	Model 1^a^		Model 2^b^		Model 3^c^	
	HR (95% CIs)	*p*-Value	HR (95% CIs)	*p*-Value	HR (95% CIs)	*p*-Value
**Primary outcomes**						
**Continuous variable**						
90-Day all-cause mortality	1.36 (1.24, 1.49)	<0.001	1.48 (1.31, 1.67)	<0.001	1.43 (1.24, 1.65)	<0.001
** Tertile**						
90-day all-cause mortality						
<3.7	1.0		1.0		1.0	
3.7–4.5	2.73 (1.41, 5.30)	0.003	2.41 (1.22, 4.77)	0.011	2.27 (1.11, 4.64)	0.025
>4.5	6.03 (3.26, 11.17)	<0.001	5.50 (2.90, 10.44)	<0.001	3.67 (1.82, 7.40)	<0.001
*p* for trend		<0.001		<0.001		<0.001
**Secondary outcomes**						
** Continuous variable**						
30-day all-cause mortality	1.32 (1.17, 1.48)	<0.001	1.41 (1.21, 1.66)	<0.001	1.33 (1.10, 1.59)	0.003
1-year all-cause mortality	1.35 (1.26, 1.46)	<0.001	1.47 (1.33, 1.63)	<0.001	1.45 (1.30, 1.62)	<0.001
** Tertile**						
30-day all-cause mortality						
<3.7	1.0		1.0		1.0	
3.7–4.5	2.42 (1.16, 5.05)	0.019	2.04 (0.95, 4.36)	0.067	2.07 (0.92, 4.68)	0.079
>4.5	4.63 (2.32, 9.20)	<0.001	3.96 (1.93, 8.12)	<0.001	2.65 (1.18, 5.93)	0.018
*p* for trend		<0.001		<0.001		0.033
1-year all-cause mortality						
<3.7	1.0		1.0		1.0	
3.7–4.5	2.77 (1.63, 4.70)	<0.001	2.26 (1.31, 3.88)	0.003	1.88 (1.07, 3.31)	0.029
>4.5	6.17 (3.77, 10.10)	<0.001	5.15 (3.09, 8.60)	<0.001	3.37 (1.93, 5.89)	<0.001
*p* for trend		<0.001		<0.001		<0.001
Length of ICU stay^d^	1.15 (0.70, 1.60)	<0.001	1.17 (0.70, 1.63)	<0.001	0.89 (0.43, 1.36)	<0.001
Length of hospital stay^d^	1.80 (1.21, 2.39)	<0.001	1.82 (1.21, 2.43)	<0.001	1.45 (0.84, 2.06)	<0.001

*HR, hazard ratio, CI, confidence interval; RA, the ratio of RDW to albumin. Models 1, 2, and 3 were derived from Cox proportional hazards regression models.*

*^a^Model 1 covariates were adjusted for nothing.*

*^b^Model 2 covariates were adjusted for age, sex, and ethnicity.*

*^c^Model 3 covariates were adjusted for age, sex, ethnicity, diastolic blood pressure, heart rate, respiratory rate, temperature, SpO2, congestive heart failure, heart valve disease, nervous system diseases, pulmonary circulation disorders, hypertension, chronic pulmonary disease, hypothyroidism, obesity, coagulopathy, white blood cell count, anion gap, bicarbonate, platelet, hematocrit, and serum chloride.*

*^d^Linear regression was used to evaluate the association between RA and length of stay. The results were expressed as β (95% CIs).*

Table 3 shows the results of subgroup analyses. The related common comorbidities were analyzed in the subgroup analysis, and no significant interaction was observed.

**TABLE 3:** Subgroup analysis of the associations between 90-day all-cause mortality and the RA level.

	No. of patients	RA level, ml/g			*p* for interaction
		<3.7	3.7-4.5	>4.5	
Age, years					0.324
<68	353	1.0	3.21 (1.08, 9.58)	8.82 (3.34, 23.29)	
≥68	354	1.0	1.61 (0.69, 3.75)	3.21 (1.44, 7.14)	
Sex, *n* (%)					0.052
Male	432	1.0	3.07 (1.26, 7.47)	9.75 (4.37, 21.78)	
Female	275	1.0	1.88 (0.69, 5.12)	2.67 (1.02, 6.97)	
Congestive heart failure					0.054
Yes	359	1.0	2.80 (1.06, 7.38)	4.10 (1.62, 10.43)	
No	348	1.0	2.05 (0.78, 5.38)	8.33 (3.63, 19.15)	
Arrhythmia					0.401
Yes	325	1.0	1.86 (0.79, 4.39)	4.20 (1.94, 9.06)	
No	382	1.0	4.44 (1.49, 13.20)	9.53 (3.36, 26.99)	
Heart valve disease					0.633
Yes	126	1.0	1.71 (0.36, 8.25)	3.07 (0.69, 13.63)	
No	581	1.0	2.90 (1.39, 6.05)	6.78 (3.45, 13.35)	
Peripheral vascular disease					0.163
Yes	76	1.0	0.77 (0.13, 4.60)	1.13 (0.24, 5.31)	
No	631	1.0	3.12 (1.52, 6.40)	7.42 (3.79, 14.53)	
Hypertension					0.921
Yes	415	1.0	2.48 (0.98, 6.29)	5.61 (2.35, 13.43)	
No	292	1.0	3.24 (1.26, 8.36)	6.59 (2.76, 15.72)	
Pulmonary circulation disorders					0.676
Yes	57	1.0	1.39 (0.14, 13.33)	2.21 (0.25, 19.78)	
No	650	1.0	2.87 (1.43, 5.74)	6.51 (3.42, 12.37)	
Chronic pulmonary disease					0.550
Yes	156	1.0	2.00 (0.53, 7.53)	3.43 (1.01, 11.72)	
No	551	1.0	2.92 (1.36, 6.28)	7.05 (3.46, 14.37)	
Coagulopathy					0.244
Yes	71	1.0	2.14 (0.43, 10.62)	2.30 (0.52, 10.19)	
No	636	1.0	2.72 (1.31, 5.65)	6.53 (3.32, 12.85)	
Electrolyte disorder					0.276
Yes	120	1.0	1.47 (0.49, 4.38)	2.38 (0.92, 6.13)	
No	587	1.0	3.45 (1.48, 8.03)	6.79 (3.02, 15.29)	
Nervous system diseases					0.679
Yes	37	1.0	1.62 (0.27, 9.72)	6.18 (1.28, 29.93)	
No	670	1.0	2.96 (1.44, 6.07)	6.32 (3.23, 12.37)	

*HRs (95% CIs) were derived from Cox proportional hazards regression models. Covariates were adjusted as in model 1 (Table 2).*

### Receiver Operating Characteristic Analysis

**FIGURE 4:**
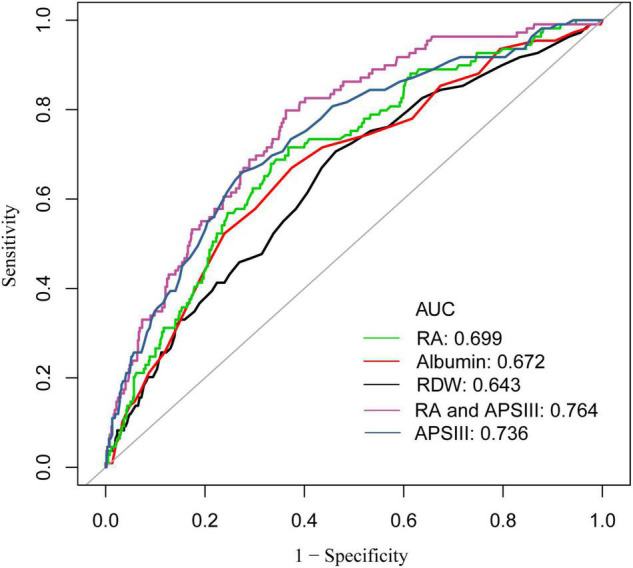
Receiver operator characteristic (ROC) curve analysis for 90-day mortality of patients with coronary heart disease, who have undergone PCI. AUC, area under the curve; RA, the ratio of red cell volume distribution width to albumin; RDW, red cell distribution width; APSIII, acute physiology score III.

Different ROC curves were respectively constructed for RA, RDW, and albumin, as well as RA combined with APSIII score (Figure 4). It was observed that the areas under the ROC curves (AUCs) (95% CIs) for RA, RDW, and albumin were 0.699 (0.647, 0.782), 0.672 (0.616, 0.728), and 0.643 (0.586, 0.699), respectively. A comparison of the AUCs proved that the ability of RA to predict the 90-day mortality was better than that of RDW or albumin. Moreover, the AUCs (95% CIs) for RA combined with SAPSIII score and APSIII score were 0.764 (0.719, 0.811) and 0.736 (0.684, 0.787), respectively (*p* < 0.05). Analysis showed that the predictive ability of RA combined with APSIII score was better than that of APSIII score alone.

### Pearson Correlation Analysis

**FIGURE 5:**
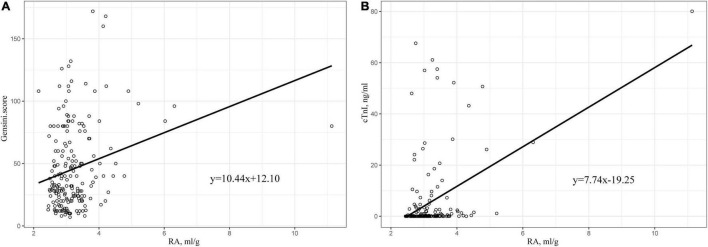
**(A)** Scatter plot of the relationship between RA and Gensini score. **(B)** Scatter plot of the relationship between RA and cTnI. RA, the ratio of red cell volume distribution width to albumin; cTnI, cardiac troponin I.

We plotted the scatter plots of Gensini score and cTnI with RA, respectively, for the data of patients in the cohort of our hospital. Results showed that both of them were positively correlated with RA (Figures 5A,B). In addition, Pearson correlation analysis was performed, and the obtained results are shown in Table 4. The correlation coefficient between Gensini score and RA was 0.254, which was better than RDW and albumin alone. A similar result was also observed in the relationship between cTnI and RA. The correlation coefficient between cTnI and RA was 0.323 (all *p* < 0.05).

**TABLE 4:** Pearson correlation analysis of the RA and clinical indicators.

	RA level, ml/g		RDW, %		Albumin, g/dl	
	*R*	*p*-Value	*R*	*p*-Value	*R*	*p*-Value
Gensini score	0.254	<0.001	0.168	0.017	−0.240	<0.001
cTnI, ng/ml	0.323	<0.001	0.152	0.034	−0.265	<0.001

*RA, the ratio of RDW to albumin; RDW, red cell volume distribution width; cTnI, cardiac troponin I. The data came from patients in the Second Affiliated Hospital of Wenzhou Medical University.*

## Discussion

This is the first study which has shown that RA is an independent risk factor for patients after PCI and high level of RA increases the length of hospital stay, even after adjusting for possible covariates. The ROC curve revealed that RA had a wonderful ability to predict all-cause mortality in patients after PCI, and its use in combination with APSIII score could enhance the predictive ability of APSIII score alone. The subgroup analysis further showed that the relationship between RA and all-cause mortality was stable. Moreover, data obtained from the cohort of our hospital showed that RA was positively correlated with Gensini score or cTnI in patients after PCI, and the correlation was stronger than RDW or albumin alone. This suggests that RA is associated with the severity of coronary artery lesions in patients with CAD.

However, the specific mechanism of the relationship between various biomarkers and mortality after PCI has not been clarified. Studies have shown that low hemoglobin levels are associated with an increased risk of all-cause mortality in patients after PCI. This may be due to the fact that low hemoglobin levels reflect to a certain extent the decline of the host’s immune response and malnutrition, which reflects the patient’s low resistance to external invasion (19). Previous studies have shown that inflammation is strongly associated with atherosclerosis and modulates the progression of coronary plaque after PCI (20–22). Chronic low-level inflammation regulates plaque formation in atherosclerosis, which subsequently leads to plaque instability and thrombosis (23–25). Evidence also suggests that reducing the classic inflammatory cascade can help reduce coronary artery-related adverse events (26).

RDW is a hematological index reflecting the volumetric heterogeneity of red blood cells (RBC) volumn size (27). RDW is automatically calculated using blood cell counters (28). Previous studies have shown that high RDW is an independent predictor of all-cause mortality in patients with CAD, even after PCI (29, 30). Inflammation affects the function of the bone marrow and inhibits maturation of RBCs, thereby leading to increased reticulocytosis and RDW. In addition, oxidative stress induces an increase in RDW by shortening RBC life span and making RBC enter the peripheral circulation prematurely, which may explain the above phenomenon (31).

Serum albumin, synthesized in the liver, is a biochemical marker of nutritional status and a major component of colloidal osmotic pressure (32, 33). Albumin is a negative acute phase protein whose synthesis rate is negatively correlated with inflammatory activity (33, 34). Experimental evidence has shown that albumin has antioxidant activity, and plays a protective role by thinning the blood and reducing oxidative stress (35–37). Several studies have shown that serum albumin level is associated with the outcomes of various diseases including cardio-cerebrovascular diseases, mainly due to malnutrition and inflammation (15, 33, 38–40). Moreover, low serum albumin concentration has been reported to be associated with adverse clinical outcomes of ischemic heart disease (41–44). A study involving more than 1,000 patients with CAD reported that reduction of albumin predicted higher all-cause mortality after PCI (45).

This study reached a similar conclusion that RA was an independent predictor of all-cause mortality in patients after PCI. RA, a combination of two classical clinical assessment parameters, showed a better prognostic value than RDW or albumin alone, as intuitively demonstrated by AUC. Moreover, RA combined with APSIII score can enhance the predictive power of the APSIII score. In addition, the data obtained from our hospital showed that RA was also positively correlated with cTnI and Gensini score. This might be attributed to the enhancement of inflammatory response in the human body by high RA level, thereby leading to the increased severity of coronary heart disease. RA, as a potential new biomarker, can be obtained quickly and easily in laboratory tests on admission, with certain advantages of simplicity. Given its low cost, good availability, and high prognostic value, RA has clinical significance for clinicians to make quick judgments on patients after PCI.

This study has several advantages. First, this is the first study to explore the association between RA and outcomes of patients after PCI. Second, the data are not only from an online database but also validated with data from our own hospital, and the results are broadly applicable. Third, the analysis fully adjusted for potential confounders that might influence the results, and the results were repeatedly validated using multiple models. Moreover, the mechanism was explored using clinical data obtained from our institution. However, the study has some limitations. First, we used a retrospective study design, and the bias of retrospective study itself cannot be ignored. Therefore, prospective studies are required to address this issue. Second, there may be selection bias in our study. Although RA is readily available in clinical practice, the loss of RA in the database is still prevalent. Due to the limitations of the database, we cannot obtain comprehensive data of the patient from the MIMIC-III database, such as the main etiology of the patient’s admission, cardiac function classification, and echocardiography results, which may have an impact on the prognosis of the patient. Therefore, further studies are needed to verify the results of this article. Next, we will collect further data and try other new methods, such as ensemble modeling and machine learning, to fully validate and refine the results of this study (46).

## Conclusion

RA is an independent risk factor for all-cause mortality in patients after PCI. The higher the RA, the higher the mortality. RA has a good predictive ability for all-cause mortality in patients after PCI, which is better than RDW or albumin alone. RA may be positively correlated with the severity of CAD in patients with CAD.

## Data Availability Statement

The original contributions presented in the study are included in the article/Supplementary Material, further inquiries can be directed to the corresponding authors.

## Ethics Statement

The studies involving human participants were reviewed and approved by the Institute of Institutional Research and Ethics of the Second Affiliated Hospital of Wenzhou Medical University. Written informed consent for participation was not required for this study in accordance with the national legislation and the institutional requirements.

## Author Contributions

All authors were involved in drafting the manuscript or revising it critically for important intellectual content, and approved the final version to be published. LW had full access to all of the data in the study and took responsibility for the integrity of the data and the accuracy of the data analysis. YW, YX, and BW contributed to the study conception and design. HX contributed to the acquisition of data. YW and YP contributed to the analysis and interpretation of data. KJ and XG contributed to excellent technical assistance with data management.

## Conflict of Interest

The authors declare that the research was conducted in the absence of any commercial or financial relationships that could be construed as a potential conflict of interest.

## Publisher’s Note

All claims expressed in this article are solely those of the authors and do not necessarily represent those of their affiliated organizations, or those of the publisher, the editors and the reviewers. Any product that may be evaluated in this article, or claim that may be made by its manufacturer, is not guaranteed or endorsed by the publisher.

## Footnotes

1
http://www.r-project.org

## Supplementary Material

Supplementary Figure 1 Curve fitting of RA and 30-day all-cause mortality in patients with coronary heart disease, who had undergone PCI.

Supplementary Figure 2 Curve fitting of RA and 1-year all-cause mortality in patients with coronary heart disease, who had undergone PCI.

Supplementary Figure 3 Kaplan–Meier survival from in 30-day all-cause mortality for patients in RA tertiles. Q1, RA < 3.7 ml/g; Q2, RA was 3.7–4.5 ml/g; Q3, RA > 4.5 ml/g.

Supplementary Figure 4 Kaplan–Meier survival from in 1-year all-cause mortality for patients in RA tertiles. Q1, RA < 3.7 ml/g; Q2, RA was 3.7–4.5 ml/g; Q3, RA > 4.5 ml/g.

Supplementary Table 1 Gensini score rule.

Supplementary Table 2 Baseline characteristics of the study population.

Supplementary Table 3 Baseline characteristics of the study population.
