# Supplementary figures and images for: The Ratio of Red Blood Cell Distribution Width to Albumin Is Correlated With All-Cause Mortality of Patients After Percutaneous Coronary Intervention – A Retrospective Cohort Study
Source: Front Cardiovasc Med. 2022 May 24;9:869816. doi: 10.3389/fcvm.2022.869816 (PMC9170887; doi:10.3389/fcvm.2022.869816)

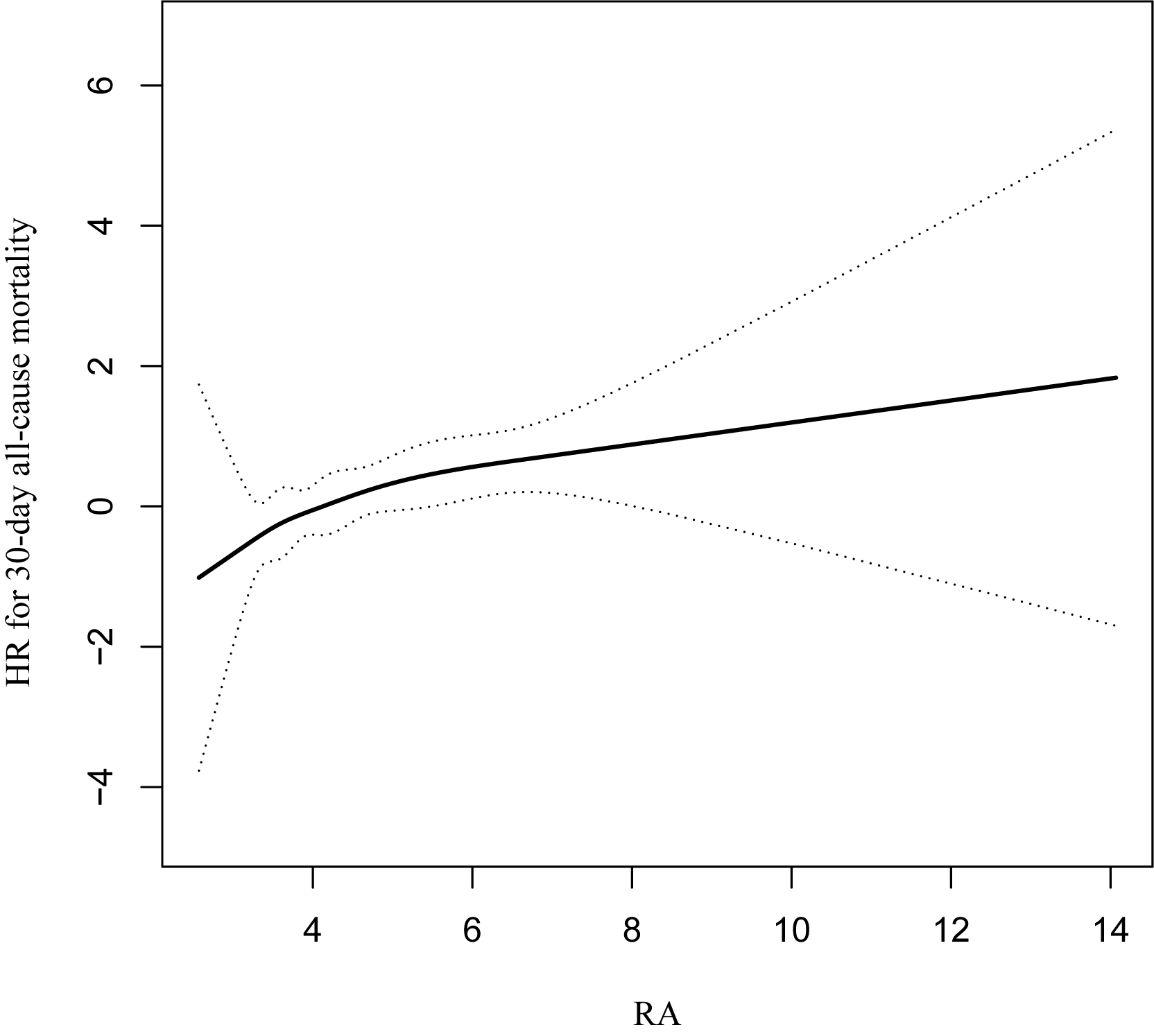

Supplement: Supplementary Figure 1 — Curve fitting of RA and 30-day all-cause mortality in patients with coronary heart disease, who had undergone PCI. [file Image_1.TIF]

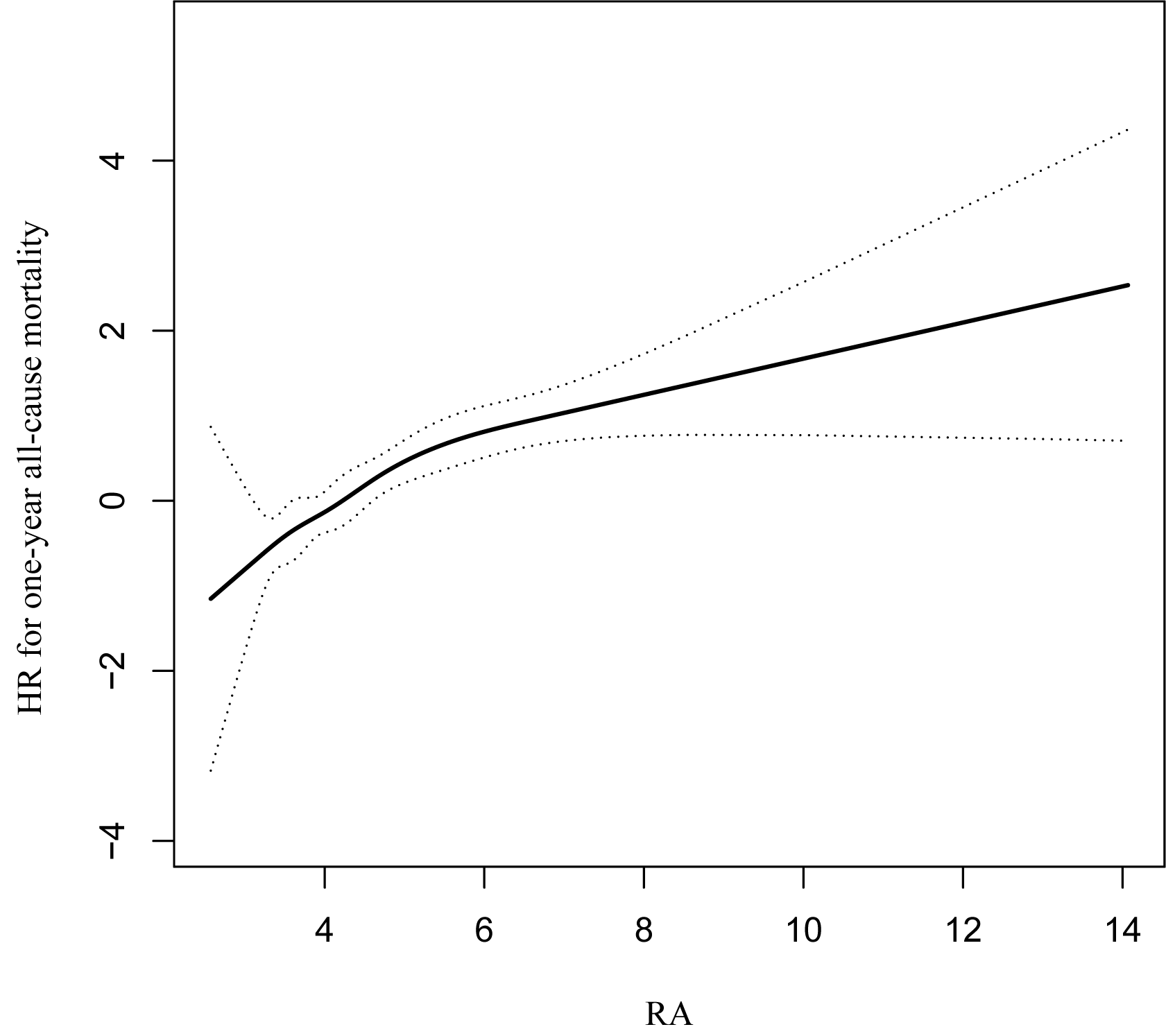

Supplement: Supplementary Figure 2 — Curve fitting of RA and 1-year all-cause mortality in patients with coronary heart disease, who had undergone PCI. [file Image_2.TIF]

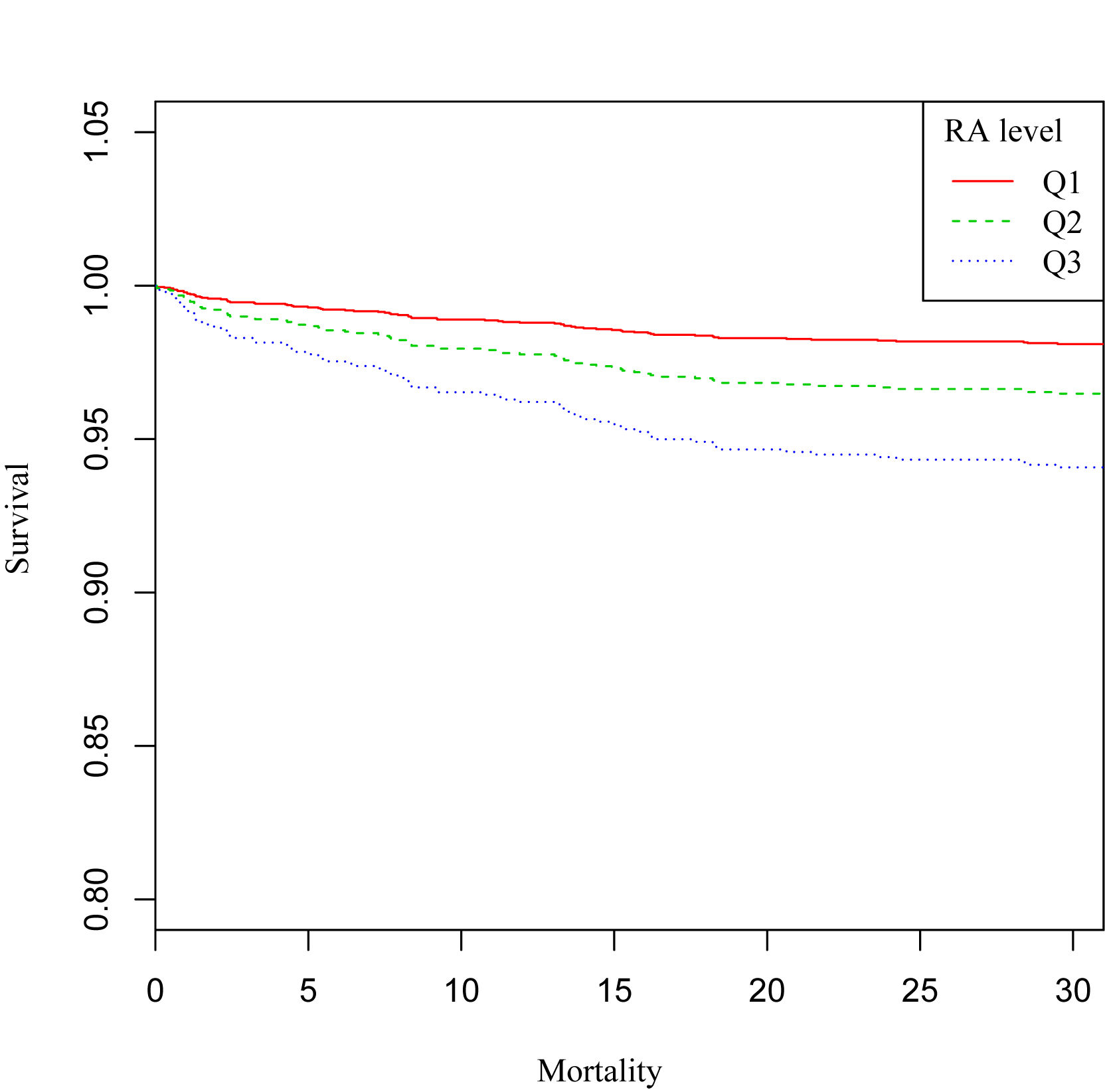

Supplement: Supplementary Figure 3 — Kaplan–Meier survival from in 30-day all-cause mortality for patients in RA tertiles. Q1, RA < 3.7 ml/g; Q2, RA was 3.7–4.5 ml/g; Q3, RA > 4.5 ml/g. [file Image_3.TIF]

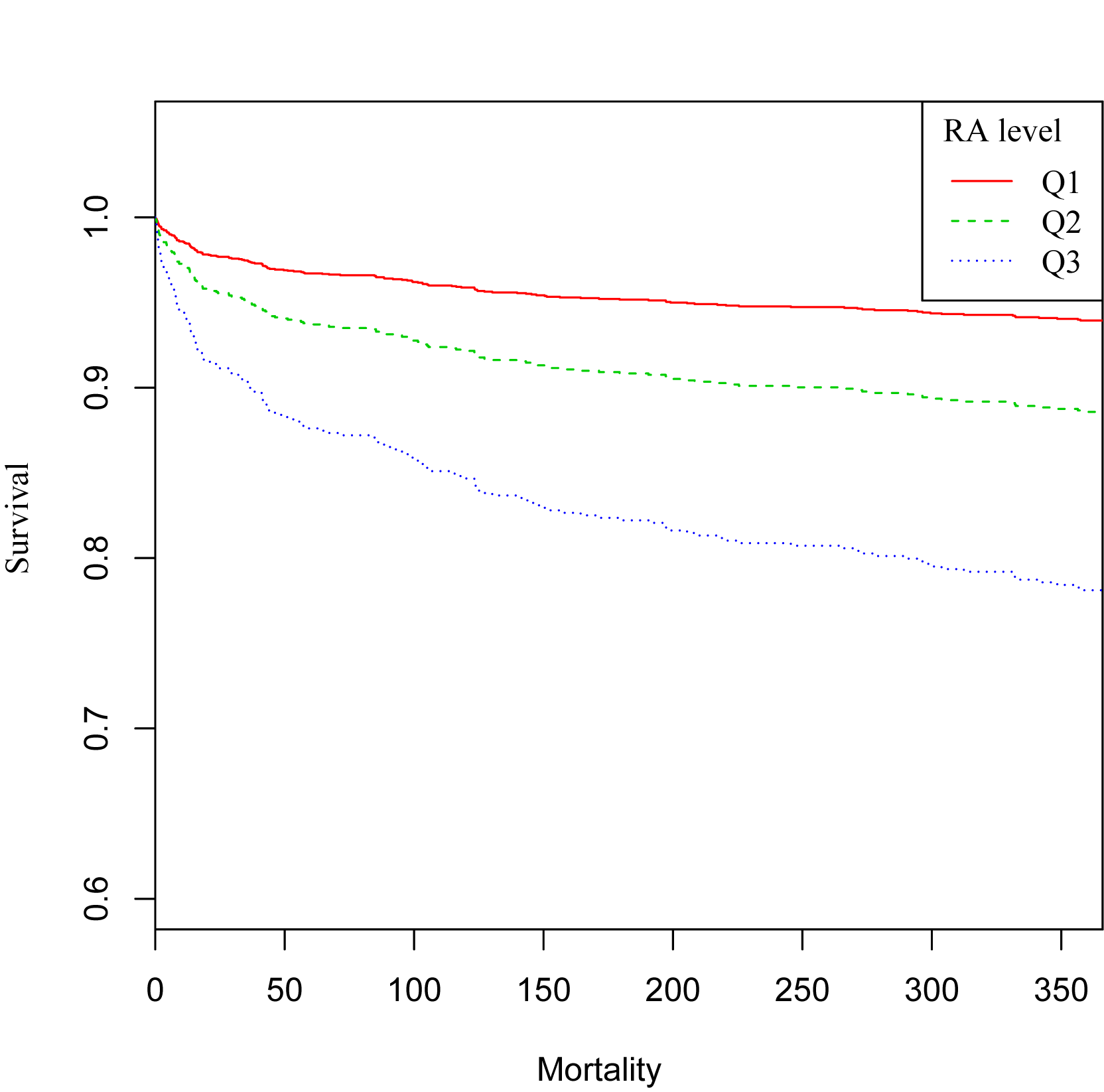

Supplement: Supplementary Figure 4 — Kaplan–Meier survival from in 1-year all-cause mortality for patients in RA tertiles. Q1, RA < 3.7 ml/g; Q2, RA was 3.7–4.5 ml/g; Q3, RA > 4.5 ml/g. [file Image_4.TIF]
