# Supplementary material for: The Ratio of Red Blood Cell Distribution Width to Albumin Is Correlated With All-Cause Mortality of Patients After Percutaneous Coronary Intervention – A Retrospective Cohort Study
Source: Front Cardiovasc Med. 2022 May 24;9:869816. doi: 10.3389/fcvm.2022.869816 (PMC9170887; doi:10.3389/fcvm.2022.869816)
Supplement: Supplementary Table 1 — Gensini score rule. [file Table_1.docx]

**Supplementary Table 1** Gensini score rule.

| Degree of stenosis | Score | Lesion site | Score |
| --- | --- | --- | --- |
| ≤25% | 1 | LM | 5 |
| 26%–50% | 2 | Proximal LAD or LCX | 2.5 |
| 51%–75% | 4 | Middle LAD | 1.5 |
| 76%–90% | 8 | Distal LAD | 1 |
| 91%–99% | 16 | Middle or distal LAD | 1 |
| 100% | 32 | RCA | 1 |
|  |  | Subbranch | 0.5 |

**Abbreviations:** LM: left main coronary artery; LAD: left anterior descending; LCX: left circumflex coronary; RCA: right coronary artery.
