# Supplementary material for: The Ratio of Red Blood Cell Distribution Width to Albumin Is Correlated With All-Cause Mortality of Patients After Percutaneous Coronary Intervention – A Retrospective Cohort Study
Source: Front Cardiovasc Med. 2022 May 24;9:869816. doi: 10.3389/fcvm.2022.869816 (PMC9170887; doi:10.3389/fcvm.2022.869816)
Supplement: Supplementary Table 2 — Baseline characteristics of the study population. [file Table_2.docx]

**Supplementary Table 2** Baseline characteristics of the study population.

| Characteristics | 90-Day all-cause mortality | | *P* value |
| --- | --- | --- | --- |
|  | Yes (n=109) | No (n=598) |  |
| **Clinical parameters** |  |  |  |
| Age, years | 73.19 ± 11.77 | 67.39 ± 13.84 | ＜0.001 |
| Sex, n (%) |  |  | 0.442 |
| Male | 63 (57.80) | 369 (61.71) |  |
| Female | 46 (42.20) | 229 (38.29) |  |
| Ethnicity, n(%) |  |  | 0.001 |
| White | 55 (50.46) | 394 (65.89) |  |
| Black | 3 (2.75) | 30 (5.02) |  |
| Other | 51 (46.79) | 174 (29.10) |  |
| **Vital signs** |  |  |  |
| SBP, mmHg | 106.28 ± 17.01 | 113.15 ± 14.73 | ＜0.001 |
| DBP, mmHg | 55.04 ± 8.60 | 60.54 ± 10.15 | ＜0.001 |
| MAP, mmHg | 73.54 ± 8.98 | 78.00 ± 9.84 | ＜0.001 |
| Heart rate, beats/minute | 86.92 ± 16.60 | 79.00 ± 14.27 | ＜0.001 |
| Respiratory rate, times/minute | 19.19 ± 3.94 | 18.46 ± 3.49 | 0.050 |
| Temperature, ℃ | 36.87 ± 0.74 | 36.78 ± 0.59 | 0.178 |
| SpO_2_, ％ | 96.83 ± 3.71 | 97.32 ± 2.01 | 0.046 |
| **Comorbidities** |  |  |  |
| Congestive heart failure, n(%) | 65 (59.63) | 283 (47.32) | 0.018 |
| Arrhythmia, n (%) | 57 (52.29) | 268 (44.82) | 0.150 |
| Heart valve disease, n (%) | 22 (20.18) | 104 (17.39) | 0.484 |
| Peripheral vascular disease,n (%) | 13 (11.93) | 63 (10.54) | 0.666 |
| Hypertension,n (%) | 55 (50.46) | 360 (60.20) | 0.057 |
| Pulmonary circulation disorders, n (%) | 8 (7.34) | 49 (8.19) | 0.763 |
| Chronic pulmonary disease, n (%) | 28 (25.69) | 128 (21.40) | 0.321 |
| Coagulopathy, n (%) | 21 (19.27) | 50 (8.36) | <0.001 |
| Electrolyte disorder, n (%) | 44 (40.37) | 76 (12.71) | <0.001 |
| Nervous system diseases, n (%) | 12 (11.01) | 25 (4.18) | 0.003 |
| Hypothyroidism, n (%) | 6 (5.50) | 49 (8.19) | 0.335 |
| Obesity, n (%) | 4 (3.67) | 26 (4.35) | 0.747 |
| **Laboratory parameters** |  |  |  |
| RA, ml/g | 5.00 ± 1.42 | 4.22 ± 1.10 | <0.001 |
| RDW, % | 15.09 ± 2.16 | 14.19 ± 1.69 | <0.001 |
| Albumin, g/dL | 3.15 ± 0.59 | 3.48 ± 0.55 | <0.001 |
| White blood cell count, 10^9^/L | 16.59 ± 7.26 | 13.57 ± 5.38 | <0.001 |
| Platelet, 10^9^/L | 246.59 ± 103.02 | 255.26 ± 87.15 | 0.354 |
| Hemoglobin, g/dL | 12.06 ± 1.84 | 12.72 ± 1.88 | <0.001 |
| Hematocrit, % | 37.68 ± 5.12 | 36.36 ± 5.50 | 0.014 |
| Serum creatinine, mg/dl | 2.41 ± 2.23 | 1.48 ± 1.46 | <0.001 |
| Serum urea nitrogen, mg/dl | 41.71 ± 28.10 | 27.28 ± 17.96 | <0.001 |
| Serum chloride, mg/dl | 105.78 ± 6.57 | 105.90 ± 4.84 | 0.821 |
| Serum sodium, mg/dl | 139.39 ± 5.67 | 139.45 ± 3.25 | 0.886 |
| Bicarbonate, mg/dl | 23.32 ± 4.58 | 24.72 ± 3.67 | <0.001 |
| Anion gap, mg/dl | 18.82 ± 4.36 | 16.31 ± 4.01 | <0.001 |
| **Scoring systems** |  |  |  |
| SAPSII | 47.50 ± 14.31 | 33.32 ± 12.98 | <0.001 |
| APSIII | 57.62 ± 23.85 | 40.05 ± 17.43 | <0.001 |
| **Length of stay in ICU** | 7.54 ± 8.55 | 4.32 ± 7.03 | <0.001 |
| **Survival time** | 21.67 ± 22.93 | 610.39 ± 645.15 | <0.001 |

**Abbreviations:** RA: the ratio of red cell volume distribution width to albumin; SBP: systolic blood pressure; DBP: diastolic blood pressure; MAP: mean arterial pressure; RDW: red cell volume distribution width ; SAPSII: simplified acute physiology score II; APSIII: acute physiology score III; ICU:Intensive Care Unit.
