# Supplementary material for: The Ratio of Red Blood Cell Distribution Width to Albumin Is Correlated With All-Cause Mortality of Patients After Percutaneous Coronary Intervention – A Retrospective Cohort Study
Source: Front Cardiovasc Med. 2022 May 24;9:869816. doi: 10.3389/fcvm.2022.869816 (PMC9170887; doi:10.3389/fcvm.2022.869816)
Supplement: Supplementary Table 3 — Baseline characteristics of the study population. [file Table_3.docx]

**Supplementary Table 3** Baseline characteristics of the study population.

| Characteristics | RA level | | | *P* value |
| --- | --- | --- | --- | --- |
|  | Low(n=67) | Middle(n=66) | High(n=67) |  |
| **Clinical parameters** |  |  |  |  |
| Age, years | 62.00 ± 13.16 | 66.45 ± 10.27 | 71.40 ± 12.43 | ＜0.001 |
| Sex, n (%) |  |  |  | 0.383 |
| Male | 46 (68.66) | 51 (77.27) | 45 (67.16) |  |
| Female | 21 (31.34) | 15 (22.73) | 22 (32.84) |  |
| **Vital signs** |  |  |  |  |
| SBP, mmHg | 137.94 ± 18.37 | 135.88 ± 21.21 | 133.72 ± 26.68 | 0.551 |
| DBP, mmHg | 81.55 ± 11.50 | 79.80 ± 12.71 | 76.37 ± 16.26 | 0.085 |
| Heart rate, beats/minute | 81.19 ± 15.75 | 75.97 ± 14.58 | 86.53 ± 19.76 | 0.002 |
| Respiratory rate, times/minute | 18.76 ± 2.12 | 19.23 ± 2.68 | 19.79 ± 3.05 | 0.083 |
| Temperature, ℃ | 36.83 ± 0.43 | 36.77 ± 0.53 | 36.61 ± 0.76 | 0.079 |
| **Comorbidities** |  |  |  |  |
| Congestive heart failure, n(%) | 5 (7.46) | 2 (3.03) | 12 (17.91) | 0.011 |
| Arrhythmia, n (%) | 5 (7.46) | 7 (10.61) | 19 (28.36) | 0.002 |
| Heart valve disease, n (%) | 4 (5.97) | 4 (6.06) | 6 (8.96) | 0.744 |
| Peripheral vascular disease,n (%) | 42 (62.69) | 48 (72.73) | 44 (65.67) | 0.450 |
| Hypertension,n (%) | 33 (49.25) | 41 (62.12) | 38 (56.72) | 0.324 |
| Cardiogenic shock, n (%) | 3 (4.48) | 7 (10.61) | 12 (17.91) | <0.001 |
| Diabetes, n (%) | 22 (32.84) | 22 (33.33) | 23 (34.33) | 0.983 |
| **Laboratory parameters** |  |  |  |  |
| RA, ml/g | 2.68 ± 0.13 | 3.05 ± 0.10 | 3.89 ± 1.08 | <0.001 |
| RDW, % | 12.34 ± 0.49 | 12.73 ± 0.57 | 13.81 ± 1.24 | <0.001 |
| Albumin, g/dL | 4.62 ± 0.32 | 4.17 ± 0.21 | 3.67 ± 0.50 | <0.001 |
| White blood cell count, 10^9^/L | 8.15 ± 3.69 | 8.84 ± 3.34 | 11.33 ± 7.02 | <0.001 |
| Platelet, 10^9^/L | 148.09 ± 68.78 | 160.11 ± 112.59 | 204.65 ± 316.01 | 0.222 |
| Hemoglobin, g/dL | 14.45 ± 1.63 | 13.92 ± 1.50 | 12.86 ± 2.07 | <0.001 |
| Serum creatinine, mg/dl | 74.66 ± 25.12 | 81.68 ± 39.93 | 103.95 ± 79.87 | 0.005 |
| Serum urea nitrogen, mg/dl | 6.45 ± 2.19 | 6.54 ± 2.34 | 37.95 ± 247.70 | 0.344 |
| Serum chloride, mg/dl | 103.20 ± 2.89 | 103.80 ± 2.98 | 104.78 ± 4.03 | 0.024 |
| Serum sodium, mg/dl | 139.81 ± 2.38 | 139.29 ± 2.80 | 139.21 ± 3.01 | 0.400 |
| cTnI, ng/ml | 5.72 ± 17.35 | 8.87 ± 22.44 | 19.86 ± 30.12 | 0.002 |
| **Scoring systems** |  |  |  |  |
| Gensini score | 36.94 ± 25.63 | 47.38 ± 33.30 | 52.49 ± 38.24 | 0.022 |
| **Length of stay in hospital** | 6.48 ± 6.54 | 7.37 ± 7.14 | 10.21 ± 9.51 | 0.017 |

**Abbreviations:** RA: the ratio of red cell volume distribution width to albumin; SBP: systolic blood pressure; DBP: diastolic blood pressure; MAP: mean arterial pressure; RA: the ratio of red cell volume distribution width to albumin RDW: red cell volume distribution width ; cTnI: cardiac troponin I.
